# Conceptual, epidemiological and methodological design aspects for the study of pre-eclampsia

**Published:** 2016-03-30

**Authors:** María Pía Monteverde, Shadia Coronel-Acosta, Eddy R Segura

**Affiliations:** 1 Escuela de Medicina, Universidad Peruana de Ciencias Aplicadas, Lima, Perú; 2 Sociedad Científica de Estudiantes de Medicina (SOCIEMUPC), Universidad Peruana de Ciencias Aplicadas, Lima, Peru

## Dear Editor:

We read with high interest the article by Alzate *et al*. 1 http://www.ncbi.nlm.nih.gov/pmc/articles/PMC4732504/, and hereby we share comments about its design, study population and statistical approach along with revisiting some key concepts of the disease.

The prevalence of preeclampsia in Colombia is 4.5% 2 and a case-control study is appropriate to investigate risk and protective factors associated in such setting and their corresponding Odds Ratios 3. However, in the population studied by Alzate *et al*., the proportion of preeclampsia is 10% (387/3,866). Under this scenario, a retrospective cohort study design is also appropriate and allows for direct estimation of incidences and relative risks could also be considered with direct estimates of relative risk. The exposure under study here (calcium prescription) is easy to measure from medical or administrative records or electronic files, therefore its comprehensive assessment in the whole population is feasible, cheap and easy to detect. Case-control studies are usually recommended when these requirements are not met for the exposure variable.

The study data was collected from two time periods. Consequently, we do not know what could have possibly changed during these years, as well as the difference in such changes between these two time periods, in the study population or in other contextual factors (health care quality, health system, regulations, physician's attitudes, medication prescription, blood pressure approaches, etc.) and how they effect on the outcome (preeclampsia) and its determinants (the way prescriptions are registered and recorded could even change over time). There is no assurance regarding the data was collected by the same team or under the same standards. This can introduce severe biases due to unmeasured confounders in both time periods. A stratified analysis for each time period can help in this regards at least partially. 

In regards to its pathophysiology and management, one of the suggested interventions is calcium supplementation. AT least one systematic review establishes the protective effect of calcium occurs at doses greater than 1 g/d of elemental calcium 4. However, Alzate *et al.,* refer to studies 5,6 that administered 600 mg/d of elemental calcium and 450 mg/d of linoleic acid in a population of women with high risk for preeclampsia on whom calcium dosage was performed to confirm the depletion of calcium before starting the supplementation. Alzate *et al.,* do not report such values or even if such dosage was performed. If this calcium depletion exists, then there is a physiological and biological plausibility grounds to attribute the positive protective effect of calcium administered. The study population belongs to the Colombian Social Security System and is likely to not have severe calcium deficiency. This is a key consideration since the effectiveness of calcium supplementation is reported in women with a pre-pregnancy deficit, so the recovery of calcium reservoirs prevents the development of preeclampsia 7.

In the statistical section we consider appropriate to take into account the variable "age" because it was different between cases and controls. However, the stratification by age is not the right approach. The reasons for this is the reduction of the sample size which leads to small numbers in specific cells and lead us to an OR of 0.0. Table 3 shows that none of the patients supplemented with CC + ALC developed preeclampsia and perhaps this is due to the stratification. A better approach is an age-adjusted multiple logistic regression to compute adjusted Odds Ratios as well as other confounders. 

Finally, the exposure variable was randomized in previous studies and the actual adherence to treatment was verified by close monitoring in each prenatal visit, questionnaires, and counting pills left in the medication container. These methods are the best to assess adherence to supplementation during pregnancy 8. This was not done by Alzate *et al.,* and questions whether the CC + ALC combination really was what prevented preeclampsia in these pregnant women. If it is not possible to measure the actual implementation (calcium intake) then we cannot assess in a valid and reliable way its effectiveness. 

In general, research about preeclampsia is important in order to better understand and manage this disease. However, we also believe all conceptual, epidemiological and methodological aspects must rigorously be taken into account in order to obtain reliable valid and generalizable results.
